# The tumour microenvironment, treatment resistance and recurrence in glioblastoma

**DOI:** 10.1186/s12967-024-05301-9

**Published:** 2024-06-06

**Authors:** Jasmine White, Madeleine P. J. White, Agadha Wickremesekera, Lifeng Peng, Clint Gray

**Affiliations:** 1 Gillies McIndoe Research Institute, Newtown, Wellington, 6021 New Zealand; 2 Department of Neurosurgery, Wellington Regional Hospital, Wellington, New Zealand; 3 Centre for Biodiscovery and School of Biological Sciences, Victoria University of Wellington, Kelburn, Wellington, 6021 New Zealand

## Abstract

The adaptability of glioblastoma (GBM) cells, encouraged by complex interactions with the tumour microenvironment (TME), currently renders GBM an incurable cancer. Despite intensive research, with many clinical trials, GBM patients rely on standard treatments including surgery followed by radiation and chemotherapy, which have been observed to induce a more aggressive phenotype in recurrent tumours. This failure to improve treatments is undoubtedly a result of insufficient models which fail to incorporate components of the human brain TME. Research has increasingly uncovered mechanisms of tumour-TME interactions that correlate to worsened patient prognoses, including tumour-associated astrocyte mitochondrial transfer, neuronal circuit remodelling and immunosuppression. This tumour hijacked TME is highly implicated in driving therapy resistance, with further alterations within the TME and tumour resulting from therapy exposure inducing increased tumour growth and invasion. Recent developments improving organoid models, including aspects of the TME, are paving an exciting future for the research and drug development for GBM, with the hopes of improving patient survival growing closer. This review focuses on GBMs interactions with the TME and their effect on tumour pathology and treatment efficiency, with a look at challenges GBM models face in sufficiently recapitulating this complex and highly adaptive cancer.

## Overview of current glioblastoma treatments

Glioblastoma (GBM) is the most aggressive primary brain tumour in adults, that accounts for more than 60% of all brain tumours in adults [1, 2]. Thought to originate from glial cells and neural stem cells (NSCs), GBM is classified as a WHO grade 4 glioma, responsible for over 15,000 deaths annually in the US alone [3–5]. A recent study in Norway determined median overall survival to be ~ 12 months, with 2- and 5-year median survivals of ~ 21% and 7%, respectively [6]. Furthermore, since prevalence increases with age, with most cases in adults between 60 and 79 years old, it is expected that the incidence of GBM will increase as life expectancy increases [7]. Despite intensive treatment with surgical resection, radiotherapy, and chemotherapy, GBM almost always recurs, often with a more aggressive phenotype [8].

**Fig. 1:**
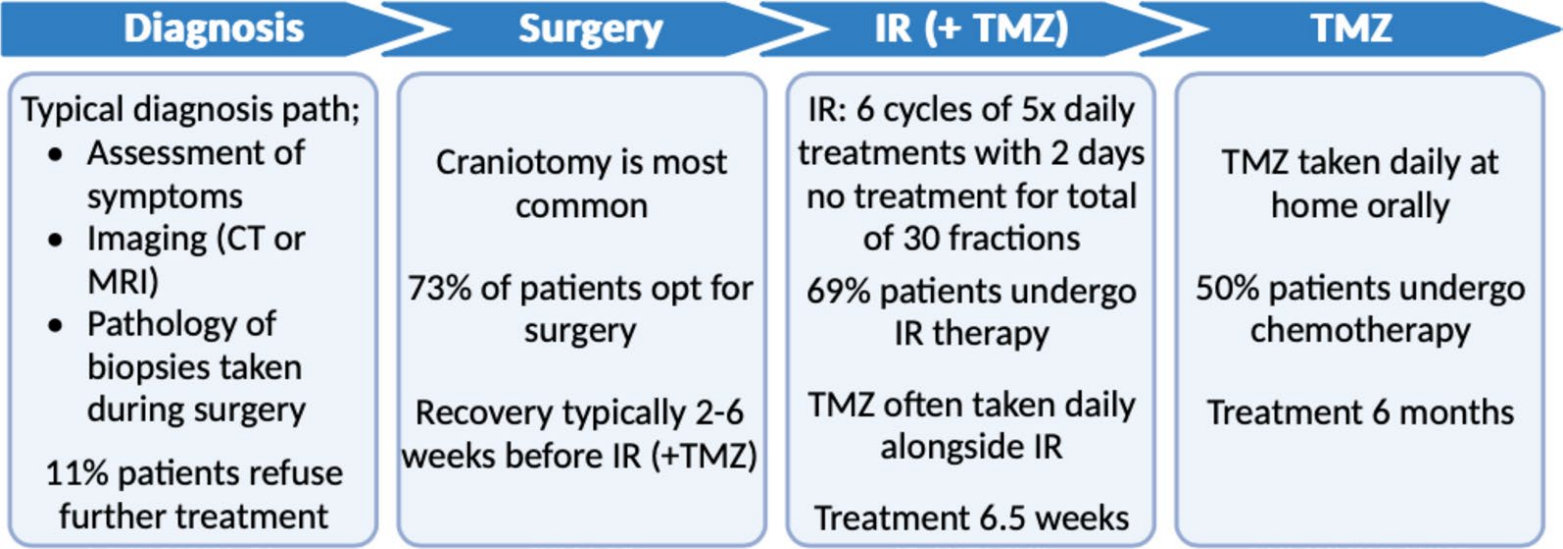
Glioblastoma standard of care typical regimen. Typical treatment regime for glioblastoma (GBM) patients with surgery followed by irradiation (IR) and chemotherapy with temozolomide (TMZ). Statistics based on the 2019 study of 100,672 GBM patients in the United States from 1998 to 2011 [15]

There are currently no known risk factors in GBM, resulting in late-stage diagnosis upon the appearance of symptoms, such as headaches, seizures, memory loss, loss of movement, cognitive impairment, and language dysfunction [9, 10]. This renders complete surgical excision of the tumour impossible due to the establishment of local serpiginous invasion and distant metastases throughout the brain which are targeted through intensive rounds of ionizing radiation (IR) and chemotherapy with temozolomide (TMZ; Fig. 1) [6]. Both surgical resection and IR are heavily limited due to the risk of damage to surrounding healthy brain tissue, with possible side effects such as paralysis, dysphasia, memory loss and personality changes outweighing the benefits of total resection [11, 12]. The use of chemotherapeutic agents is also limited due to limited infiltration of drugs through the highly selective blood brain barrier (BBB) [13]. Currently, the only therapeutic agents approved for GBM treatment are TMZ, carmustine and bevacizumab (BEV), with little success in other agents through clinical trials due to failure passing the BBB, a long-acknowledged limitation specific to brain cancer treatment, and the inherent resistance of highly interconnected GBM tumours [13, 14].

Tumour-treating fields (TTFields), the non-invasive delivery of alternating electric fields with frequencies ranging from 100 to 300 kHz, is gaining traction in the clinic for GBM treatment [16, 17]. TTFields are currently approved for newly diagnosed GBM with concomitant TMZ, impressively improving overall survival by 4.9 months in the EF-14 study (NCT0096409) in 2015, and recurrent GBM as a monotherapy, improving quality of life in the EF-11 study (EF-11; NCT00379470) in 2011, in the USA, Canada, Hong Kon, Japan, Europe, Israel, and Australia [18, 19]. The biophysical force exerted on dipoles by these electrical fields were shown to increase cell permeability via increased pore formation in the cell membrane of cancerous cells, with no effect on healthy cells, increasing TMZ efficiency [16, 17]. Other effects of TTFields include altered cell cycle, decreasing proliferation, increased autophagy, reduced migration, reduced cell metabolism, delayed DNA repair and enhanced stress during DNA replication, observed with increased IR efficacy in GBM cell lines [20, 21]. This data is promising for the future of GBM therapies, with the potential to increase drug delivery selectively to GBM cells and induce strong pro-inflammatory effects, opening opportunities for immunotherapies which previously showed low efficacy in clinical trials [20].

Another therapy approved for use in GBM patients is the implantation of carmustine (Gliadel® wafer), an alkylating agent-soaked tissue, into the resection cavity [22]. However, this is not a universal practice due to concerns over toxicity and post-surgery complications such as cerebral edema, seizures, intracranial hypertension, cerebral fluid leaks, intracranial infections, and healing abnormalities with only marginal increases to patient survival [22, 23]. Hence, considering the effects to surrounding healthy brain tissue is crucial in the development of therapies to combat this lethal and debilitating disease.

Further difficulties with treatment arise through the intrinsic ability of GBM to resist chemo- and radiotherapy, with over 50% of patients not responding to treatment with TMZ [24]. Consequently, GBM almost always recurs within 18 months with a more aggressive phenotype, growing and invading into surrounding brain tissue at an increased rate [8, 25]. Furthermore, treatment for recurrent GBM is limited to the same therapies that proved unsuccessful at irradicating the primary tumour, with the addition of BEV which showed no survival benefit in clinical trials [26]. Therefore, patients treated for recurrent GBM have significantly lower survival rates as the tumour itself is seeded from treatment-resistant cells, often growing, and invading at a faster rate into the already damaged surrounding brain tissue [8].

Recent research reveals interactions between GBM and the surrounding tumour microenvironment (TME), including stromal cells and the immune population, to contribute to tumour aggressiveness and treatment evasion [14, 27]. Furthermore, GBM treatment itself has been observed to modify GBM tumours and the surrounding TME, with evidence of surgery, radiation and chemotherapy altering tumour interactions, increasing tumour growth and invasion, as well as contributing to increased therapy resistance [27]. This research further highlights the urgent need for better treatments for both primary and recurrent GBM. Recent advances in cerebral organoid models, incorporating aspects of the human brain TME, offer promising prospects for GBM research, with the potential of greater translational success of research into the clinic.

## Glioblastoma heterogeneity

Despite intensive research, the devastating prognosis of GBM has remained mostly unchanged for 30 years, with only a slight increase of ~ 2 months when the standard of care protocol was enacted in 2005 [28–30]. GBM mechanisms of dysregulation remain perplexing, and consequently difficult to treat, due to GBM’s heterogeneity, complex interactions in the surrounding microenvironment, and ability to maintain subpopulations of cells within the tumour with varying genotypes and phenotypes [2, 31]. These specific abnormalities, along with therapeutic limitations, including drug transfer across the BBB, have led to barriers in GBM research and resulted in the failure to improve treatment options.

**Fig. 2:**
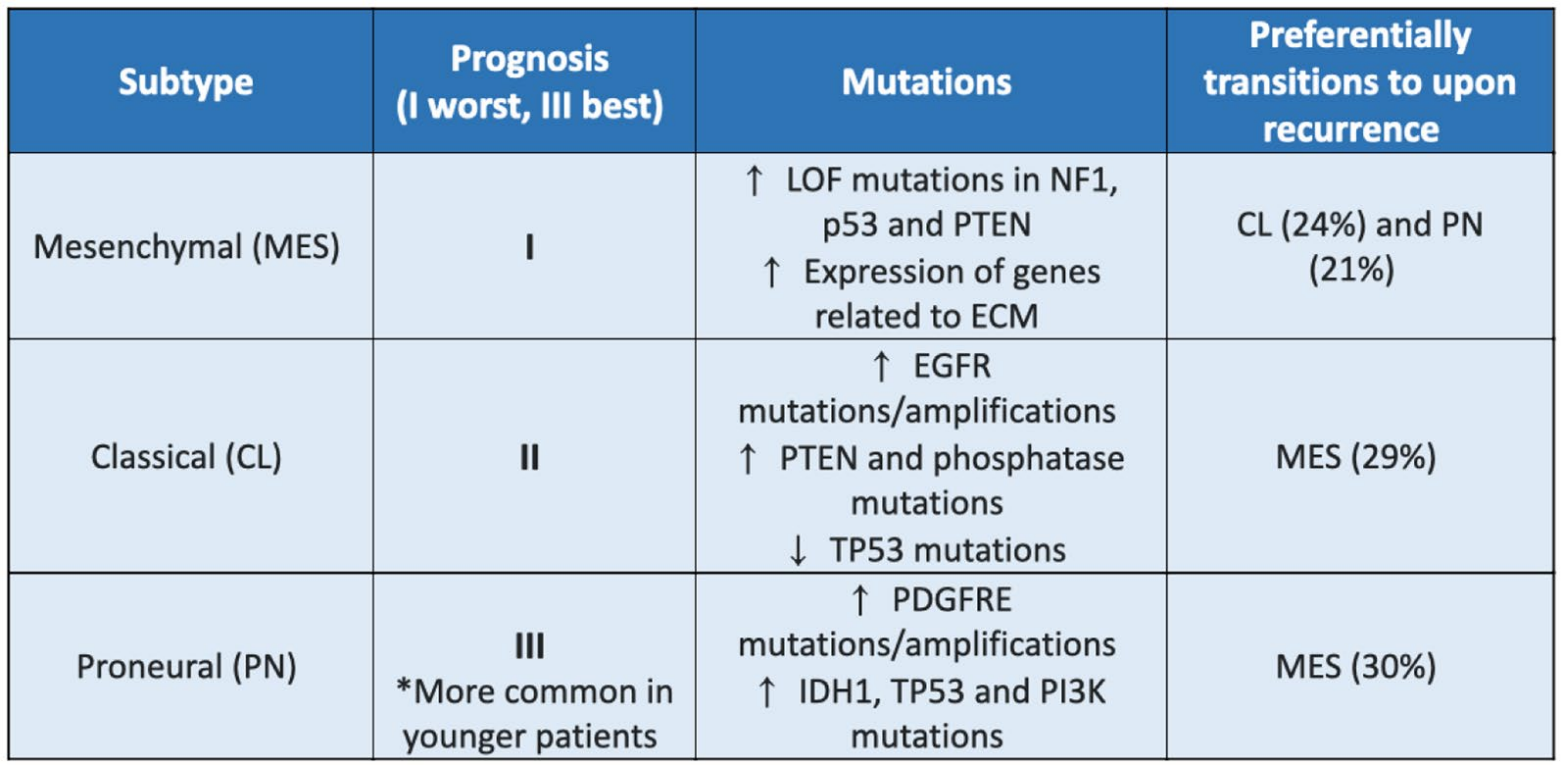
Glioblastoma subtype classification. The three main subtypes of glioblastoma, MES, CL, and PN, are classified by differing mutation frequencies [37]. CL and PN subtypes are most commonly observed to switch to the more aggressive MES upon recurrence, whilst MES tumours preferentially transition to the CL subtype, which is classed as being more aggressive than the PN subtype [33]

GBM is known as a highly heterogeneous cancer, thought to be the root cause of therapeutic resistance, exhibiting both inter- (between patients) and intra- (within patient tumours) tumoural heterogeneity [32]. For example, varying levels of *O*^6^-methylguanine-DNA methyltransferase (MGMT) promoter methylation are observed between patients, with hypermethylation reducing chemoresistance, increasing patient survival by 6.8 months [6]. GBM patients are often ascribed to one of the three subtypes: classical (CL), mesenchymal (MES), and proneural (PN), depending on the dominant mutational signature of the biopsy tissue (Fig. 2) [32, 33]. However, this purely inter-tumoural classification fails to consider the variable, and interchangeable, expression of the three subtypes throughout a single patient’s tumour [32, 33]. The presence of transcriptionally distinct cells harbouring different microenvironments throughout single tumours exhibit varying responses to treatments, invariably resulting in the survival of subgroups of tumour cells, is becoming widely accepted [28, 32–36]. Recent immunofluorescence of specific biomarkers identified 8 different clusters in GBM tissue, further categorized into five pathophysiologically relevant groups which did not exclude each other [32]. Furthermore, single cell RNA sequencing (ScRNA seq) revealed GBM cells to exhibit plasticity, demonstrating their capacity to transition between states [33]. This is evident in the observed switching between subtypes of patient tumours (Fig. 2), initially subtyped as primary tumours and then again on the onset of recurrence [33, 36]. Hoogstrate et al. also observed alterations to stromal cell and innate immune cell distributions within the TME, suggesting TME reorganisation to influence, or be influenced by, GBM subtype switching [33]. Hence, any one treatment specific to a subtype of GBM is insufficient to treating a GBM tumour, as the survival of other subtypes, existing within different microenvironments, will eventually result in recurrence.

### Glioma stem cells

Glioma stem cells (GSCs) are a subpopulation implicated in tumorigenesis and tumour propagation that are established throughout GBM tumours but account for as few as 1% of cells in a tumour [24, 38, 39]. With the ability to retain stemness and properties very close to neural stem cells, GSCs have the capacity to self-renew and generate many cells of differing lineages, thereby sustaining intratumoural heterogeneity [38–40]. This has been observed by the presence of varying amplifications of receptor tyrosine kinases (RTKs) throughout patient tumours and their metastases [31, 41]. The maintenance of many subpopulations permits GBM to interact with different cell types, allowing them to adapt to and induce changes within the TME [31]. These functionally distinct tumour cells and consequently differing responses may permit the survival of certain subpopulations within GBM upon treatment, increasing the likelihood of persistence of the tumours growth and dissemination [31]. Hence, GSCs are becoming widely accepted as regulators of GBM growth, maintenance, invasion, treatment evasion, and recurrence, sustaining GBMs inherent aggressive nature [29, 40, 42].

GSCs have also been observed to display increased resistance to chemotherapeutics and IR [43, 44]. This resistance is thought to arise through multiple mechanisms, including the upregulation of anti-apoptotic genes, such as BCL-xL, and the activation of proteins involved in DNA damage repair through phosphorylation, such as Chk1 [44–46]. For these reasons, there is a large focus on specifically targeting these tumour-initiation, treatment-resistant GSCs [29]. However, since they exist in a complex microenvironment which influences the maintenance of stemness, it is crucial to also consider targeting the TME [47].

## The tumour microenvironment

In recent years, the understanding of tumour growth has shifted from a cell-centric view, in which the tumour is treated as a single entity, to a dynamic and ever-changing accumulation of complex interactions within the TME [14, 48]. Since different tumours originate from different cell types and grow in different environments, these interactions vary between tumour types and even subtypes [49]. Furthermore, as tumours grow and evolve, their environment is altered, allowing tumour cells to evolve as is seen within the three subtypes of GBM; MES, CL, and PN, which are able to transition into each other in response to TME remodelling (Fig. 2) [28, 33].

Due to dynamic interactions between GBM and the TME, the TME is also heavily influenced by changes within the tumour due to altered cell-to-cell contacts, metabolic products, oxygen availability and changes to the extracellular matrix (ECM) [14]. For example, high expression and secretion of the ECM glycoprotein, tenascin-C (TNC), by GSCs at the leading edge creates a TME favouring tumour cell migration away from the tumour mass [50, 51]. Furthermore, invading GBM cells have been observed to utilise its surrounding TME to regulate shifts in their own osmotic potential via Cl− and K+ channels, allowing invasion into smaller spaces [50, 52]. Hence, there is a complex interplay between the behaviours of GBM and surrounding, noncancerous brain cells that must be understood in order to efficiently target and prevent GBM growth and invasion.

GBM’s TME is comprised of many noncancerous cells, including astrocytes, oligodendrocytes, neurones, resident immune cells, tumour-infiltrating circulating immune cells, and vascular endothelial cells, as well as noncellular components, such as ECM components, paracrine signalling molecules, exosomes and chemical factors such as acidity and oxygen availability [14, 27, 53]. This heterogenous mix of cells gives rise to many direct cell-to-cell interactions and therefore the possibility of many different signalling pathway attenuations [14, 27]. A recent study revealed different tissue states (e.g., a reactive/inflammatory state vs. a cellular/proliferative state) to be comprised of different proportions of specific cell types, giving rise to different metabolic signatures [54]. Furthermore, since the rate of GBM growth commonly overcomes angiogenesis, nutrient supply and oxygen availability across a tumour can vary considerably, resulting in altered metabolism across the tumour [14]. Therefore, it is clear that in order to improve our knowledge of the drivers of GBM genesis, growth, and invasion, greater insights into mechanisms and effects of TME-tumour interactions are required.

### Astrocytes

Astrocytes are the most abundant cells within the brain, making up approximately 50% of brain cells and are crucial to maintaining brain homeostasis [62]. Astrocytes are classified as glial cells that can be heterogeneous in different brain regions due to varying signalling [62]. NSC-derived astrocytes are one of the three cells believed to give rise to GBM, along with NSCs and oligodendrocyte precursor cells (OPCs) [5]. Different astrocyte phenotypes perform different roles, including maintaining the BBB, regulating inhibitory and excitatory transmission, and supporting tissue repair at damaged sites [62]. Following brain injury, local astrocytes undergo astrogliosis, generating reactive astrocytes with increased transcription of components required for increased growth, including growth factors, signalling receptors, and cell adhesion proteins to repair the site of injury [62]. GBM has the ability to hijack this response, initiating the transformation of astrocytes into tumour-associated reactive astrocytes (TARAs), in turn promoting GBM growth and invasion through a myriad of signalling attenuation via direct signalling, paracrine signalling, and structural enhancement [62]. For example, TARAs secrete factors, such as TGF-β and IL-6, increasing GBM proliferation and invasion, respectively, whilst also providing protrusions that GBM cells can invade along [62]. Furthermore, various studies have correlated the enrichment of TARAs to an inflammatory/reactive tumour state, increased GBM cell survival, promotion of the evasion of apoptosis, GSC population invasiveness, and responses to hypoxia including the promotion of angiogenesis [54, 62].

**Fig. 3:**
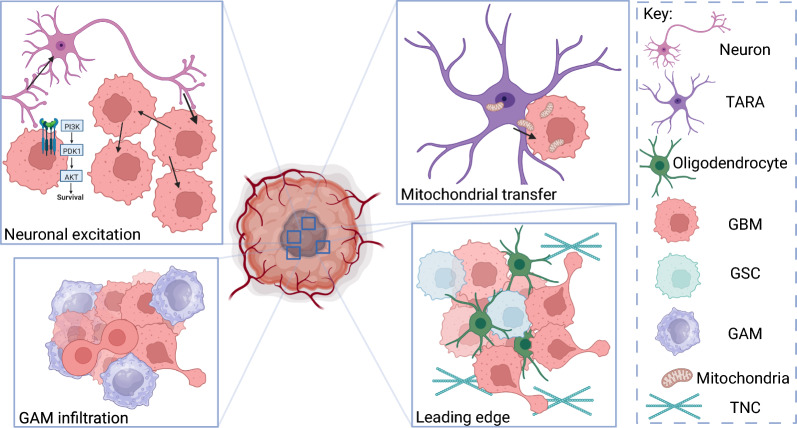
Interactions with stromal cells in a glioblastoma tumour. Neuron-derived neuroligin-3 can increase PI3K-mTOR pathway attenuation, whilst neuronal excitement can be propagated across glioblastoma (GBM) cell networks via gap junctions [55–57]. Tumour-associated astrocytes (TARA’s) can transfer mitochondria to GBM cells via gap junctions. The infiltration of glioma-associated microglia/macrophages (GAMs), and increased presence of oligodendrocytes and glioma stem cells (GSCs) with increased secretion of tenascin-C (TNC) by GSCs at the leading edge, regulates increased proliferation and invasion of GBM [51, 58–61]

New mechanisms of TARA-GBM interconnectivity are continuously being revealed. For example, Watson et al., recently identified the horizontal transfer of mitochondria from TARAs to GBM cells, via tumour microtubules, facilitating increased GBM metabolism thereby promoting cell cycle progression and enhancing tumorigenesis (Fig. 3) [58]. Additionally, since mitochondria are involved in the regulation of apoptosis and ferroptosis, amongst other cell death pathways, their accumulation in GBM cells may correlate an increased resistance to cell death and therefore therapies [58, 63]. Other studies observing gap junctions between TARAs and glioma cells have suggested the exchange of toxins and small molecules, decreasing the risk of programmed cell death, thereby promoting cell health [14]. Much evidence supports that astrocytes within the GBM microenvironment are integral to the generation of a tumour-progressive environment through many mechanisms, potentially facilitating increased inflammation, decreased susceptibility to metabolic stress, and increased migration.

### Neurons

Neuronal activity has long been implicated in promoting GBM growth, with well characterised pathways such as PI3K-mTOR activation in GBM cells following neuroligin-3 secretion from neurons (Fig. 3) [55]. Moreover, electrical synapses from neurons (pre-synaptic) to tumorous GBM cells (post-synaptic) were recently identified in Venkataramani’s lab, where excitatory activity was observed to correlate increased growth and invasion (Fig. 3) [56, 57]. Furthermore, it has been observed that gliomas can integrate into and remodel neuronal circuits, increasing neuronal excitability and therefore tumour growth [57]. Since GBM is known to have high interconnectivity, with gap junctions between glioma cells throughout the tumour, electrical impulses can propagate broadly (Fig. 3), inducing many outcomes, including increased microtubule turnover to facilitate invasion [14, 56]. Hence, research suggests these bidirectional interactions between neurons and GBM to generate the ideal environment for sustained tumour growth, with higher neuronal activity correlating a reduced survival rate. This was also observed in GBM patients performing language tasks, with higher degrees of functional connectivity between GBM and normal brain correlating reduced performance and decreased survival [57]. The ability of GBM tumours to remodel neuronal circuits, exciting and aiding tumour growth has thus far been observed only in GBM, perhaps describing how seizures, which increase neuronal activity, result in a worsening patient prognosis [56].

### Oligodendrocytes

Similar to astrocytes, oligodendrocytes are glial cells with many roles in maintaining cerebral homeostasis, including neuronal activity regulation and the support of axon myelination [59]. Oligodendrocytes are more commonly detected at tumour border niches (Fig. 3), including the invasion front and resection border, suggesting their potential influence in both invasion and recurrence [59]. Recent research has confirmed oligodendrocytes to enhance GBM migration, but not proliferation, through the release of cytokines such as angiopoietin-2, which, when blocked, has been shown to reduce migration and prolong patient survival [64, 65]. Furthermore, oligodendrocytes have been observed to promote angiogenesis in GBM, further supporting GBM tumorigenicity [66]. Additionally, Hide et al., attributed oligodendrocytes in the promotion of GSC niches, with the transfer of oligodendrocyte precursor-cultured medium to GBM cells inducing stemness as well as chemo- and radioresistance [60]. Hence, oligodendrocytes may be involved in tumour recurrence at tumour borders following resection by supporting GSCs proliferation and tumour cell invasion.

### Immune population

The largest immune population in GBM are microglia and tumour-infiltrating macrophages, accounting for as many as 30–50% of cells within GBM’s microenvironment [61, 67, 68]. Once associated with GBM’s environment, microglia and macrophages are referred to as glioma-associated microglia/macrophages (GAMs) which produce a range of factors including anti-inflammatory cytokines, tumour promoting factors, angiogenesis promoting factors, and disrupt metabolism [14]. The presence of GAMs is associated with increased glioma proliferation, migration, ECM degradation, angiogenesis, and T-cell apoptosis (Fig. 3) [61, 69, 70]. More recent research revealed the bidirectional interaction between GAMs and tumorous glioma cells to induce an immunosuppressive and pro-tumorigenic environment, with GAMs inducing TME remodelling, suppressing T-cell anti-tumour activity and inducing the transition to a MES-like state [33, 61, 71–73]. Hambarzumyan et al. reported a positive correlation between GAM presence and poor prognosis with worse overall survival [74, 75].

Many immune components have been implicated in shaping GBMs TME. For example, myeloid-derived suppressor cells (MDSCs) have been elucidated in sensing and adapting to modifications of nutrients, oxygen and inflammatory signals, promoting tumour growth [76]. MDSCs have been observed to inhibit glucose uptake, impairing the metabolism of activated T-cells, which is further compromised by the deprivation of glucose in the TME due to the preferential glucose metabolism of GSCs [76, 77]. The inherent resistance of GSCs to immune responses has been extensively elucidated in numerous studies [77, 78]. Mechanisms underpinning GSCs reduced susceptibility to immune surveillance include the expression of checkpoint inhibitors, the downregulation of antigen presentation molecules and the induction of immunosuppressive myeloid cells, including GAMs [77, 79]. Furthermore, a recent study observed the percentage of c-Met and FasL (GSC markers) to the frequency of GAMS, regulatory T cells (Tregs) and tumour-infiltrating lymphocytes (TILS), further elucidating GSCs in immune evasion in GBM [78].

Other components of the immune population include TILs, of which T cells are the primary component but constitute < 0.25% of total cells, natural killer (NK) cells, and neutrophils [80]. Neutrophil abundance in GBM’s TME have been associated with an immunosuppressive role and consequent poor survival [14, 80]. Interestingly, the more aggressive mesenchymal subtype of GBM is enriched for neutrophils and M2 macrophages, mediating increased immunosuppression, promoting GBM growth and development [80, 81]. On the other hand, natural killer cells, whose role is to mediate the removal of pathogens and stressed cells, have been observed at low levels in GBM tumours [14]. The secretion of TGFβ from GBM cells as well as stromal cells within the GBM TME has been observed to reduce NK cell activation, preventing the removal of the highly stressed GBM cells [14, 82].

Due to inherent immune evasion, many immune based therapies that have proved beneficial, such as immune checkpoint inhibitors, in other complex cancers have rendered limited effects in GBM. However, the first phase III trial for dendritic cell vaccines (DCVs) published promising data for glioblastoma immunotherapies in 2022, showing a significant increase in patient survival [83]. Moreover, research suggests DCVs loaded with antigens targeting GSCs may increase OS [84]. Another immunotherapy approach emerging as a promising therapy for GBM is chimeric antigen receptor (CAR) T cell, also indicating promising data in preclinical and clinical trials [85–87]. Interestingly, CAR-T therapy has been shown to be efficient against GSCs, with the targeting of B7-H3 showing anti-tumour activity against GSC-enriched neurospheres and differentiated GBM cell lines [85, 86]. Furthermore, since CAR-T therapy does not rely on the endogenous immune response and utilizes immune trafficking to cross the BBB, this therapy may circumvent immunosuppressive and CNS-entry issues encountered by other therapies [86]. Further research detailing mechanisms of GBM and the immune population interactions may shed light on how to improve the efficiency and longevity of existing immunotherapies to facilitate their translation into the clinic.

### Chemical environment

Generated by differing metabolic rates within tumour cells and distance from blood vessels, fluctuating soluble factors such as nutrients, ATP, O_2_ (Fig. 3), Ca^2^+, reactive oxygen species (ROS), and H+ concentrations throughout the tumour are observed [88, 89]. Each of these factors can impact cellular function and thereby effect tumour heterogeneity, growth and treatment resistance [88, 90]. Hypoxic TMEs have been extensively linked to tumour aggressiveness, including the promotion of GSC self-renewal and the upregulation of stem cell factors in non-stem cell tumour populations, thereby promoting de-differentiation, as well as increased cell invasion [32, 91]. Due to increased stem-like phenotypes within hypoxic niches and the dependency of IR on ROS generation for DNA damage, GBM cells within hypoxic niches are naturally more chemo- and radioresistant [88, 92]. Furthermore, increased ROS levels in the TME have been observed to promote immune suppression, tumour growth and therapy resistance [88]. Oxidative stress has also been implicated in mediating cell type conversion, such as fibroblasts to myofibroblasts, commonly known as cancer-associated fibroblasts (CAFs) due to their promotion of tumour aggressiveness through many faceted approaches, including increased proliferation, invasion, and inflammation [88, 93].

Lactate accumulation, resultant of increased anaerobic glycolysis in GBM tumours, generates acidic regions within the tumour associated with increased therapy resistance [88, 94]. Acidosis has also been linked to supporting the aggressive phenotype of GBM cells with structural remodelling of the ECM, encouraging GBM growth and invasion, and supporting GSCs via increased expression of GSC markers [95, 96]. Moreover, acidosis may contribute to the immunosuppressive environment within GBM by reducing the infiltration and activity of T cells and blocking the cytotoxic activity of natural killer cells, further promoting GBM’s persistence [97]. Hence, the metabolic state and consequent microenvironment is a crucial component in tumour biology and will have effects on treatment efficiency.

## Considering the TME in GBM research

GBM rarely colonise outside the CNS (< 0.5%), with up to 80% of recurrent tumours occurring within 2.5 cm of the initial resection cavity post-operation, with 77% of patients exhibiting recurrence at the original tumour edge [28, 53]. Furthermore, 60 of the 88 patients who had no residual tumour at post-operative MRI were shown to display tumours within 2 cm of the primary tumour site at recurrence [98]. This highlights the importance of the interactions between GBM cells and the TME and its role in maintaining and promoting tumour survival and growth. With compiling evidence suggesting the TME’s involvement in the promotion of subtype plasticity, GSC stemness, and therapeutic resistance, research is starting to focus on targeting not only dysregulated tumour pathways, but also the TME. Due to this shift in understanding, many labs are focusing on improving models to better recapitulate in vivo TME components with the hopes to further our understanding of tumorigenic-promoting TME–GBM interactions and better predict clinical responses to therapeutic intervention.

### TME-dependent treatment resistance

Research shows evidence that interactions between GBM and the TME can induce resistance to both chemo- and radiotherapy. Therefore, a greater understanding of the TME-mediated signalling pathways responsible for generating therapy resistance is undoubtedly crucial to reducing recurrence. It is common practice to remove, and irradiate, GBM tumours with a 2 cm margin into the surrounding tissue to prolong the onset of recurrence since GBM commonly recurs at the border of the resected cavity [59, 99]. There are many TME-driven interactions mitigating signalling towards a more aggressive tumour phenotype. This tumour-promoting environment has also been observed to regulate treatment resistance, thereby facilitating recurrence of more aggressive, treatment-resistance GBM.

GSCs themselves are inherently chemo-radioresistant due to high plasticity with increased expression of DNA repair proteins and anti-apoptotic proteins as well as an increased metabolic capacity [100]. Multiple components of the TME promote GSC lineages, including oligodendrocytes at the tumour border, thereby directly affecting tumour response to both chemo- and radiotherapy [59, 60]. Hypoxic niches, typical to the GBM environment due to rapid proliferation and outgrowth of capillaries, as well as macrophages have also been observed to increase GSC markers, correlating increased chemo- and radio-resistance [28, 60, 101].

The immune microenvironment of GBM is known to be ‘hijacked’ by GBM tumour cells. Tumour-associated GAMs have been observed to induce the transition to an MES-like state, which is associated with increased resistance to both chemo- and radiotherapy [72, 102, 103]. Furthermore, the expression of genes involved in detecting tumour cells and signals and initiating immune-driven responses were observed to be downregulated in microglia derived from mice GBM, whilst genes promoting tumour spread were upregulated [104]. Research has shown that adjuvant immunotherapy against specific antigens can reduce survival in TMZ-resistant GBM cells, suggesting a role for GBM’s immune microenvironment in the contribution to TMZ-controlled cell death evasion [105–107]. However, whilst immune-targeting therapies, such as monoclonal antibodies, have been successful in other cancers, they have not successfully progressed through GBM clinical trials due to low efficacy [108].

**Fig. 4:**
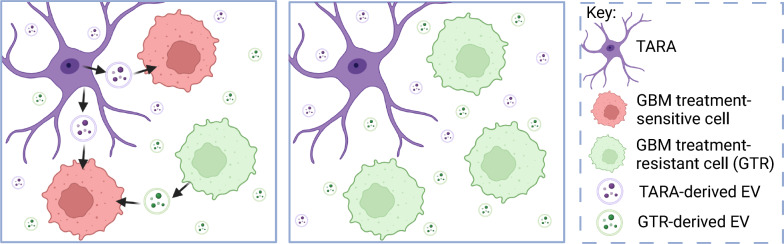
Extracellular vesicles can regulate glioblastoma treatment resistance. Tumour-associated reactive astrocyte (TARA) and glioblastoma (GBM) treatment-resistant cell (GTR) derived extracellular vesicles (EVs) can induce treatment resistance in glioblastoma treatment-sensitive cells [109, 110]

The transfer of lipids, nucleic acids, and proteins via extracellular vesicles (EVs) have been observed to promote chemoresistance. For example, the transfer of MGMT mRNA from TARAs to GBM cells via EVs promotes a TMZ-resistant phenotype (Fig. 4), whilst EVs transferring miR-1246 have been observed to induce M2 macrophage polarisation, thereby promoting immunosuppression in hypoxic environments [109, 111]. The transfer of molecules via EVs from tumour cells to non-tumour cells, and tumour-associated stromal cells to other stromal cells, has also been implicated in promoting a pro-tumorigenic TME [24, 105]. Research has also shown exosomes from TMZ-resistant GBM cells to increase survival of TMZ-sensitive cells (Fig. 4), confirming EVs to encourage a tumour promoting, treatment-resistant environment by altering cellular function [110]. Furthermore, hypoxic stress has been observed to alter EV cargo, release, and uptake, promoting gliomagenesis [24, 112]. This suggests that hypoxic regions within a GBM tumour harbour increased EV transfer, potentially increasing chemo- and radioresistance through molecular, transcriptional, and translational modifications [24, 88, 105]. Hence, high interconnectivity within a GBM tumour may enable therapy resistance through a many faceted approach.

The TME also affects the metabolic burden on tumour cells. Astrocytes can donate mitochondria to GBM cells via tumour microtubules [58]. Increased ATP generation rates may supply energy demands for increased growth, movement, and upregulation of repair pathways, such as the DNA-damage repair pathway, increasing the tolerance of cancer cells to chemo- and radiotherapy. Furthermore, the accumulation of mitochondria is associated with GBM chemo- and radioresistance, with the transfer of mitochondria between CAFs and GBM cells under cytotoxic stress suggested to aid the evasion of apoptosis [113].

Hypoxic regions within a tumour are associated with increased IR resistance due to decreased sensitivity to ROS and consequent apoptosis evasion [25, 88]. Lactate accumulation in hypoxic regions is suggested to generate highly acidic regions which potentially neutralise the ROS produced in response to irradiation [88]. Since treatment resistance depends on a cell’s ability to maintain redox homeostasis, it could be speculated that sharing ROS throughout a network of cells, via tumour microtubules or EVs, could reduce the concentration of ROS within a single cell and increase resistance to radiotherapy.

Due to the aggressive nature of recurrent GBM, with a prognosis of only months reducing the number of patients eligible for surgery, tissues to research recurrent GBM are sparse. It is clear that the tumour-TME interactions contributing to therapy resistance must be further researched and perturbed to improve treatment efficiency. Further routes to improve treatment efficiency include targeting tumour-associated stromal and immune cells alongside conventional treatments to reduce resistance and increase tumour death, thereby reducing the incidence of recurrence.

### Treatment-dependent TME adaptations

Since interactions between the TME and GBM cells can induce growth and invasion, large changes to either of these niches can result in further adaptations to the TME, tumour cells and their interactions [27]. Currently there are no effective treatments specifically adapted for recurrent GBM, leaving patients to rely on the standard-of-care treatment for primary GBM, which provide very limited success. Furthermore, recurrent tumours have been shown to be governed by different molecular pathways and are functionally distinct to a patient’s primary tumour [28, 114]. Typically, patients diagnosed with recurrence have a median survival of 3 to 14 months, depending on treatment regimens [6]. Hence, it is crucial to gain a better understanding of the cells that drive recurrence and their interactions with the altered TME that drive a more aggressive phenotype and develop therapies that specifically target recurrent pathways.

It has long been accepted that surgical resection can affect the growth rate of the residual tumour. Studies in mammary tumours revealed tumour doubling time to decrease, with an observable increase in tumour growth rate, also observed in a 2013 study with GBM in mice following resection with a twofold increase of the proliferative marker Ki-67 [114, 115]. Damage to the TME resulting from surgical resection has been observed to stimulate reactive astrocytes, microglia, and macrophages, consequently increasing GBM proliferation and migration [27, 116]. Hingten’s group later revealed temporal and spatial alterations in reactive astrocytes, known to play a key role in tumour proliferation and migration, on the periphery of the tumour post-resection [116]. They went on to reveal injury to astrocytes to promote tumour proliferation and migration [116]. This reactive astrocyte-dependent progression was recently determined to be governed by the secretion of Cxcl5, a cytokine known to drive cancer progression, into the TME [117].

IR is known to result in long-term brain damage, with side effects associated with high doses including haemorrhage, cognitive decline, and neurodegeneration [118]. Hence, radiation therapy is majorly limited by dose, often resulting in the prevalence of tumour cells that have evaded IR-induced death. GBM treatments have high failure rates, even at high doses, resulting in significant molecular alterations within the prevailing tumour cells, thus participating in tumour relapse [88].

The build-up of ROS and oxidative stress following IR has been associated with alterations to GBM cell survival pathways and EV-induced cellular reprogramming towards pluripotency, adding to therapy resistance [119]. IR has also been observed to alter networks, with changes to cell adhesion, the ECM, and normal neuron-glia networks, allowing aggressive invasion through a weakened environment with reduced tension [25, 27]. Hence, IR primes the TME for tumour relapse, assisting increased growth and invasion of treatment resistant GBM cells following senescence.

IR has also been shown to induce increased VEGF expression and vascular remodelling, causing vascular depletion, hypoxia, neo-angiogenesis and increased cell migration and invasion [25, 120]. Increased BBB permeability has also been observed, increasing immune infiltration and recruitment of macrophages at the tumour leading edge, also associated with increased tumour invasion and aggressiveness [25, 121]. However, the VEGF monoclonal antibody, BEV, shows no significant improvements to patient survival in clinical trials, yet is still used to treat recurrent GBM patients due to decreased cerebral edema and improved neurologic symptoms [26, 122].

Interestingly, long-term chemotherapy with TMZ and BEV has also been observed to induce migration and invasion in glioma cells, with changes observed in the microenvironment such as immune suppression [20, 27]. TMZ has also been shown to alter GBM gene expression, with an increased expression of CDK1, promoting entry to mitosis, and stem-like markers in recurrent tumours [8, 123]. Prolonged exposure of GBM to TMZ was also shown to result in decreased expression of TLR4 in GBM cells, promoting TLR4 suppression in GAMs, contributing to increased tumour migration and immune evasion [124, 125]. Furthermore, similar to IR, TMZ therapy is linked to increased EV release with tumour-promoting cargo alterations, including an accumulation of molecules related to cell adhesion and invasion [125]. Hence, TMZ treatment is associated with alterations to both GBM and TME proteomes, promoting a more aggressive phenotype in surviving populations, ultimately reducing recurrent GBM sensitivity to treatments, thereby reducing patient prognosis.

Though it is apparent the standard of care therapies used to treat primary GBM themselves have detrimental effects to tumour growth, potentially triggering the onset of a more aggressive phenotype that drives recurrence, these therapies are integral to overall patient survival and quality of life. Patients following the standard Stupp protocol had a median survival of 16.1 months compared to 1.8 months in patients receiving only surgical resection with no post-operative treatment [6]. Research is actively searching for pathways that facilitate treatment resistance, including interactions with the TME, that could be targeted to improve treatment efficiency. Encouragingly, research suggests the activation of a strong immune response to TTFields in primary GBM [20]. Further research is required to develop therapies specifically for the treatment of recurrent GBM which is undoubtedly altered by, and acquires resistance to, primary therapies.

### GBM models

**Fig. 5:**
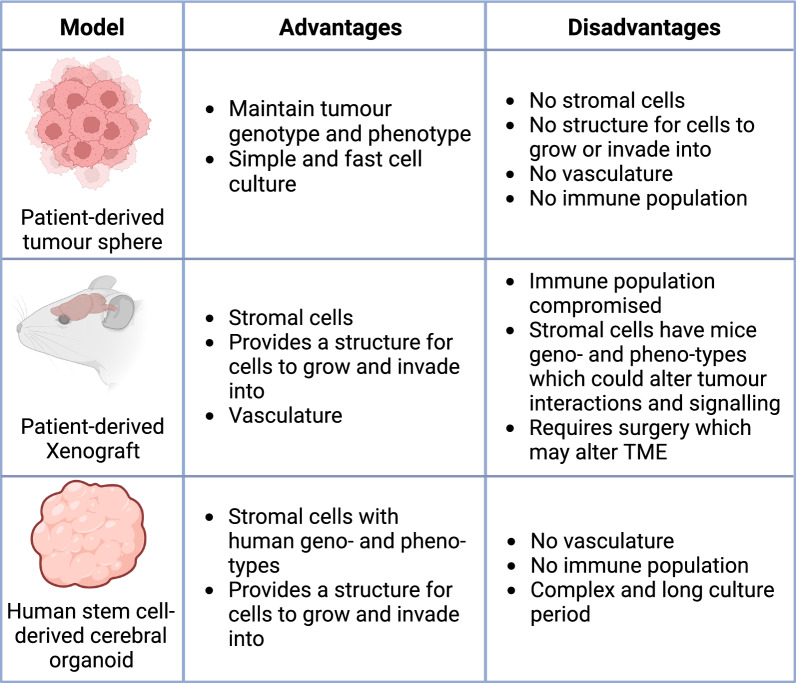
Glioblastoma models advantages and disadvantages. Advantages and disadvantages of three-dimensional tumour spheres, the xenograft mouse model and cerebral organoids which are increasingly becoming the basis for complex glioblastoma models

The heterogeneity and requirement for complex models that better recapitulate the complex GBM microenvironment is arguably partly responsible for the failure to translate basic research into successful treatments in the clinic. Historically, 3D spheroid models, derived from GBM tumour cells, have been the most used model for GBM. Whilst these models closely represent in vivo GBM characteristics, such as morphology and proliferation, they fail to include the tumour architecture and TME-tumour interactions (Fig. 5) which are an ever evolving and crucial component to GBM tumour biology [14, 28].

Efforts to improve GBM models for more clinically relevant research involve the incorporation of the TME, such as vascularisation, components of the immune system and brain-resident cell types including neurons and astrocytes. As with many other disease models, animal models have been extensively used in GBM research, offering valuable insights into glioblastoma biology, therapeutic efficacy, and potential adverse effects. Commonly used syngeneic mouse models, which rely on the expansion of established tumour cell lines in vitro, including the mouse derived GL261, SMA-560 and SB28 cell lines [126]. The major benefit to these models is the ability to implant tumours in immunocompetent mice, allowing insights into interactions between the immune and tumour microenvironment [126, 127].

Due to differing origins, it’s important to consider the advantages and limitations of each model, such as the choice to study invasion and immunosuppression in SMA-560 models over SB28 models due to poor representation of immunogenicity [126]. Conversely, SB28 may be better suited to studies of the TME than GL-261 models which have observed dissimilarities to the TME of human GBM [126, 127]. However, since these cell lines are derived from a single GBM tumour, they fail to address the vast heterogeneity that is observed between patient tumours. The importance of this is highlighted by the vast number of clinical trials that have failed in patients following successful mice preclinical studies [126, 127]. The most used animal model, the patient-derived xenograft (PDX) model, which is generated by the implantation of patient derived GBM tissue or tumour spheres in immunodeficient mice, fails to incorporate the full immune complement of GBM (Fig. 5) [126]. Furthermore, there are substantial differences between the mice and human brains including structure and functional organisation (Fig. 5) [28, 128]. These evolutionary genetic differences may explain complications in the translations of findings from mouse models into human-safe therapeutics [129].

Other models becoming widely used in GBM research are the in vitro 3D organoid models, which incorporate human TME components and preserve tumour heterogeneity, thereby better recapitulating GBM tumour biology (Fig. 5) [130]. There are different organoid models, with groups constantly implementing novel approaches to further improve models. These include neoplastic cerebral organoids (neoCOR), patient-derived organoids (PDO), and cerebral organoid-GBM co-cultures (GLICO) models [130, 131]. Each model utilises different techniques, for example, the GLICO model is generated through the co-culture of cerebral organoids with patient-derived tumour spheres, whilst neoCOR models involve the introduction of genetic mutations in a small subset of cells within a cerebral organoid to generate tumorigenesis [29, 132]. Hence, there are advantages and disadvantages to each model, with uses in different areas of GBM research. For example, the GLICO model may help determine TME-tumour interactions facilitating invasion, whilst the noeCOR model could be used to understand TME-tumour interactions in early GBM development [130, 133]. Whilst these models are increasingly implemented into GBM research, there are similar drawbacks with lacking vasculature and immune components (Fig. 5). Nevertheless, research is ongoing to further develop these models.

## Conclusions and future perspectives

It is widely accepted that GBM’s TME is intricately linked to tumour survival, progression, and treatment resistance. However, due to its heterogeneity and ability to adapt, it is difficult to model GBM’s TME in vivo. The future of GBM treatment depends on the development and utilisation of reproducible models incorporating all aspects of the TME to gain greater insights into the interconnectivity and subsequent treatment evasion mechanisms in GBM patients. With the development of new organoid models, which are increasingly becoming the standard for GBM research, the trajectory for discovering treatments with a greater chance of successfully progressing through clinical trials and significantly improving patient outcomes is exciting.

## Data Availability

Not applicable.
